# Thymol-Loaded Polymeric Nanoparticles Improve the Postharvest Microbiological Safety of Blueberries

**DOI:** 10.17113/ftb.61.02.23.7595

**Published:** 2023-06

**Authors:** Syarifah Ab Rashid, Woei-Yenn Tong, Chean-Ring Leong, Wen-Nee Tan, Chee-Keong Lee, Mohd Razealy Anuar, Siew-Hway Teo, Siti Khalida Abdull Lazit, Jun-Wei Lim, Nur Amiera Syuhada Rozman

**Affiliations:** 1 Universiti Kuala Lumpur, Branch Campus Malaysian Institute of Chemical and Bioengineering Technology, Lot 1988 Kawasan Perindustrian Bandar Vendor, Taboh Naning, 78000 Alor Gajah, Melaka, Malaysia; 2 School of Biological Sciences, Universiti Sains Malaysia, 11800 Minden, Penang, Malaysia; 3 Universiti Kuala Lumpur, Institute of Medical Science Technology, A1-1, Jalan TKS 1, Taman Kajang Sentral, 43000 Kajang, Selangor, Malaysia; 4 Chemistry Section, School of Distance Education, Universiti Sains Malaysia, 11800 Penang, Malaysia; 5 Bioprocess Technology Division, School of Industrial Technology, Universiti Sains Malaysia, 11800 USM, Minden, Penang, Malaysia; 6 HICoE-Centre for Biofuel and Biochemical Research, Institute of Self Sustainable Building, Department of Fundamental and Applied Sciences, Universiti Teknologi PETRONAS, Seri Iskandar, 32610 Perak Darul Ridzuan, Malaysia; 7 Department of Biotechnology, Saveetha School of Engineering, Saveetha Institute of Medical and Technical Sciences, Chennai 602105, India

**Keywords:** blueberries, microbiological safety, nanoparticles, postharvest treatment, thymol

## Abstract

**Research background:**

The presence of *Yersinia enterocolitica* on raw food products raises the concern of yersiniosis as most of the berries are consumed raw. This is a challenging issue from the food safety aspect since it could increase the occurrence of foodborne diseases among humans. Thus, it is crucial to implement an effective sanitation before the packaging.

**Experimental approach:**

This study aims to synthesize and characterize thymol-loaded polyvinyl alcohol (Thy/PVA) nanoparticles as a sanitizer for postharvest treatment of blueberries. Thy/PVA nanoparticles were characterized by spectroscopic and microscopic approaches, prior to the analyses of antimicrobial properties.

**Results and conclusions:**

The diameter size of the nanoparticles was on average 84.7 nm, with a surface charge of −11.73 mV. Based on Fourier transform infrared (FTIR) measurement, the Thy/PVA nanoparticles notably shifted to the frequency of 3275.70, 2869.66, 1651.02 and 1090.52 cm^-1^. A rapid burst was observed in the first hour of release study, and 74.9 % thymol was released from the PVA nanoparticles. The largest inhibition zone was displayed by methicillin-resistant *Staphylococcus aureus* (MRSA), followed by *Y. enterocolitica* and *Salmonella typhi*. However, amongst these bacteria, the inhibition and killing of *Y. enterocolitica* required a lower concentration of Thy/PVA nanoparticles. The treatment successfully reduced the bacterial load of *Y. enterocolitica* on blueberries by 100 %.

**Novelty and scientific contribution:**

Thymol is a plant-based chemical without reported adverse effects to humans. In this study, by using the nanotechnology method of encapsulation with PVA, we improved the stability and physicochemical properties of thymol. This nanoparticle-based sanitizer could potentially promote the postharvest microbiological safety of raw berries, which may become an alternative practice of food safety.

## INTRODUCTION

Fruits and vegetables are the most common vehicles implicated in foodborne disease outbreaks (*1*). In 2010, the US Department of Agriculture revealed that about 18.9 billion pounds of fresh fruits and vegetables are wasted annually due to spoilage (*1*). This accounts for 19.6 % of all edible food lost in the USA (*2*). In terms of food safety, fresh fruits and vegetables are considered a high risk for microbial contamination. Microbial spoilage of these sources is usually due to the raw material and postharvest processing equipment contact (*1*). Besides human contact with fruits and vegetables during picking, water used for washing during pre- and post-harvesting processes can also cause microbial contamination.

*Yersinia enterocolitica* is a coccobacillus-shaped Gram-negative bacterium. It is a psychrophilic bacterium that can grow and survive at low temperature (4 °C). It is frequently isolated from rodents, domestic animals, and water contaminated by these animals (*3*). Fruits and vegetables can be contaminated by faeces of these domestic animals or the person handling the products (*4*). Besides, the imported fruits and berries have been brought into connection with the increased threat of yersiniosis as most of the berries are consumed raw (*5*). Due to this risk factor, in the USA alone, the pathogen causes 640 hospital admission cases, 117 000 illnesses and 35 deaths (*6*). Several outbreaks have been associated with frozen berries since *Y. enterocolitica* grows well at refrigeration temperature (*7*). Thus, sanitizers are used to prevent microbial growth on berries.

Chemical sanitizers are commonly used for postharvest treatment of fruits and vegetables to restrain the growth of spoilage bacteria. They include sodium hypochlorite, iodine, hydrogen peroxide and quaternary ammonium compounds (*8*). However, these chemicals can cause skin irritation, mucous membrane damage, or carcinogenic and mutagenic damage (*9*). They also affect ecological system once released into air, water and soil. More importantly, chemical sanitizers also cause food deterioration such as loss of nutritional quality, colour and flavour (*8*). Due to the consumer demand for safe and good quality food, chemical sanitizers are often substituted with natural alternatives (*9*). However, nonchemical sanitizers are less effective than chemically synthesized compounds due to their poor stability (*10*). The natural compounds also interact negatively with food components, which affects the food quality (*8*).

Thymol (5-methyl-2-isopropylphenol, C_10_H_14_O) is a bioactive compound present in thyme (*Thymus vulgaris*) oil. Moreover, it holds a Generally Recognized as Safe (GRAS) food ingredient status (*11*). This compound is a colourless crystalline substance that provides strong flavour, pleasant odour and has a strong antiseptic property. However, owing to its poor stability and high volatility, thymol application is restricted in food systems. Furthermore, the compound has low water solubility at neutral pH (*12*). Its pungent taste and smell also interfere with the protein and fat present in food, which causes poor palatability (*11*). All these shortcomings limit the usage of thymol as an antimicrobial agent in the food system.

Nanotechnology can be applied to improve the stability and physicochemical properties of materials, including thymol. Nanoparticles are particulate substances or solid particles within a 2-100 nm size range (*13*). The nanoscale size influences the physicochemical properties of natural compounds, so the nanoparticles usually exhibit better biological activities (*14*, *15*). Thus, in this study, nanotechnology was applied to synthesize and characterize thymol with polyvinyl alcohol (PVA) as an encapsulant material. The antimicrobial efficiency of synthesized nanoparticles was evaluated on food spoilage microorganisms. More importantly, we also determine the efficiency of thymol nanoparticles as fruit sanitizer to inhibit the growth of *Y. enterocolitica* on frozen berries.

## MATERIALS AND METHODS

### Synthesis of thymol nanoparticles

Thymol nanoparticles were synthesized using polyvinyl alcohol (PVA) as encapsulant (*16*). Firstly, 0.3 g of thymol (Solarbio, Beijing, PR China) was mixed in 5 mL of 25 % ethanol (Thermo Fisher, Waltham, MA, USA). Next, 50 mL of 2 % Pluronic F127 (Sigma-Aldrich, Merck, St. Louis, MO, USA) were mixed with thymol solution using silent crusher (Heidolph, Schwabach, Germany). Then, 50 mL of 2 % PVA solution (Sigma-Aldrich, Merck) were added and mixed at 8944×*g* for 5 min until a clear solution was observed. Then, the solution was kept in a freezer (-80 °C), prior to freeze-drying (Labconco Freeze Dry System, Missouri, MO, USA). The freeze-dried nanoparticles were kept in a desiccator prior to use, and these particles are called Thy/PVA nanoparticles in this study. A control was provided by replacing the thymol solution with ethanol and these particles are called blank nanoparticles. For antimicrobial assays, the nanoparticle was dissolved in 20 % Tween 20 to a desired concentration and strained through a filter (0.22 μm pore size; Millipore Sigma, Billerica, MA, USA) prior to use.

### Determination of nanoparticle morphology

The size and shape of the developed nanoparticles were determined *via* transmission electron microscopy (TEM; Philips CM12; Eindhoven, The Netherlands). To fix the sample for microscopic observation, a droplet of Thy/PVA nanoparticle solution was dropped on a carbon-coated copper grid. It was followed by a drop of uranyl acetate stain. The sample was left to dry at an ambient temperature, prior to microscopic observation.

### Surface charge

To determine the surface charge of thymol nanoparticle, dynamic light scattering (DLS) was measured in clear disposable zeta cell with zeta analyser (Malvern Zetasizer Nano-ZS90; Malvern Instruments Ltd., Malvern, UK). The test temperature was set at 25 °C.

### Encapsulation efficiency

The encapsulation efficiency of nanoparticles is defined by the amount of the bioactive ingredient (thymol) encapsulated in the nanoparticles (*17*). Thy/PVA nanoparticles were first dissolved in ethanol at a ratio of 1:10 (*m*/*V*). The sample was subjected to ultrasonic bath for 20 min to release the thymol into the solvent phase. The amount of released thymol was determined by gas chromatography system (Auto System XL model; PerkinElmer, Waltham, MA, USA) equipped with fused silica capillary column (30 m×0.32 mm i.d., 0.25 µm film thickness). Hydrogen was applied as the carrier gas with a flow rate of 1 mL/min. A total of 0.2 µL of sample was subjected to the chromatographic system. The injection and detector temperature were set up at 250 °C. The column temperature was set at 100 °C for 1 min and programmed to increase at a rate of 15 °C/min to 240 °C, where it was kept for 1 min. Thymol standards were prepared at the concentration range of 62.50 to 1000 µg/mL to construct a calibration curve for determination of the thymol concentration in the sample. The encapsulation efficiency was calculated according to the following equation:


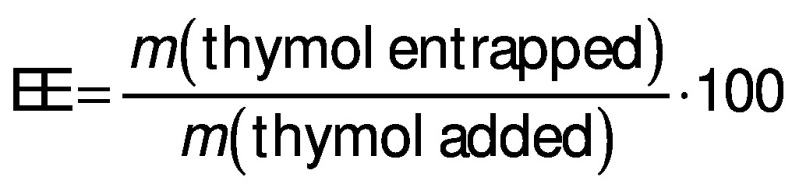
 /1/

### Fourier transform infrared analysis

Fourier transform infrared (FTIR) spectroscopy was performed to study the chemical interactions between thymol and its encapsulant. The FTIR spectra of Thy/PVA nanoparticle, PVA and unencapsulated thymol were determined using Thermo Fisher Scientific Nicolet iS10 FTIR spectrometer. The FTIR spectra were recorded in the range of 4000-600 cm^-1^ at room temperature (25 °C).

### Thymol release property

Firstly, 100 mg of Thy/PVA nanoparticles were placed in phosphate buffer solution (10 mL, pH=7.4). The solution was placed in an incubator shaker (Model BJPX-N; Biobase, Shandong, PR China) and agitated at 1×*g* and 37 °C. Then, 500 µL of the test sample were withdrawn at fixed time points, specifically at 1, 2, 4, 8, 24, 48 and 96 h. The amount of thymol released in the test medium was analysed with gas chromatography as per protocol described for encapsulation efficiency study. The experiment was performed in three replicates at different times. The results were presented as the amount of thymol released against time to study the drug release behaviour of the nanoparticle system.

### Test bacteria

The antimicrobial efficiency of Thy/PVA nanoparticles was tested on foodborne microorganisms. The test microorganisms included both Gram-positive (*Bacillus cereus*, *Staphylococcus aureus* and methicillin-resistant *S. aureus* (MRSA)) and Gram-negative bacteria (*Escherichia coli*, *Salmonella typhi* and *Yersinia enterocolitica*). These bacteria were previously isolated from contaminated food samples (*17*, *18*). The inoculum size of bacterial suspensions was adjusted to 10^8^ CFU/mL by comparing the turbidity with 0.5 McFarland standard, prior to the experiment.

### Kirby-Bauer test

Kirby-Bauer test was done to screen the antimicrobial activity of the nanoparticles (*18*). A total of three test substances were used, namely Thy/PVA nanoparticle (10 mg/mL), positive control (chloramphenicol 100 µg/mL) and negative control (blank nanoparticles dissolved in 20 % Tween 20). First, the bacterial suspension was swabbed on the surface of Müller-Hinton agar (Merck) using a sterile cotton swab. Then, 20 µL of test substance were pipetted on the sterile paper discs (6 mm diameter) and placed on the surface of the agar. The plates were kept at 37 °C for 24 h and then the diameters of inhibition zones were measured. The test was done in triplicate.

### Broth microdilution assay

The minimal inhibitory concentration (MIC) and minimal bactericidal concentration (MBC) of Thy/PVA nanoparticles were determined on the test bacteria that showed significant susceptibility in the Kirby-Bauer test (*18*). The assay was performed in a flat bottom 96-well cell culture plate (Biologix Research Co, Kansas, MO, USA). The inoculum was prepared by adding 1 mL of the bacterial suspension into 9-mL sterile double strength Müller-Hinton broth (Merck, New Jersey, NJ, USA). Then, 100 µL of Thy/PVA nanoparticles at different concentrations (1.25 to 20.00 mg/mL) were added to obtain 200 µL as the final volume in each well. The final concentrations of Thy/PVA nanoparticles were set from 0.63 to 10.00 mg/mL. For growth control, 20 % Tween 20 solution was added to the inoculated broth. For sterility control, Thy/PVA nanoparticles at various concentrations (0.63 to 10.00 mg/mL) were added to sterile broth. Then, the plate was kept at 37 °C for 24 h. After that, 20 µL of 0.2 mg/mL *p*-iodonitrotetrazolium violet salt were pipetted into each well. The plate was then stored at 37 °C for 1 h in the dark. The colour changes from yellow to pink indicate the bacterial growth. The MIC was the lowest concentration of Thy/PVA nanoparticle that retards the bacterial growth. To determine the MBC, one loopful of sample from each well was suitably streaked on the Müller-Hinton agar plate. The plates were kept at 37 °C for 24 h and the viability of the test bacteria was monitored. MBC was fixed as the lowest concentration of Thy/PVA nanoparticle needed to kill the test bacterium. The assay was done in triplicate.

### Bacterial growth curve

*Y. enterocolitica* was used for this assay to investigate the effect of Thy/PVA nanoparticle concentration on bacterial growth. This bacterium was selected as it had the lowest MIC and MBC values. A total of 100 μL of bacterial inoculum was inoculated into 5 mL of sterile Müller-Hinton broth. To obtain a final volume of 10 mL, 4.9 mL of Thy/PVA nanoparticle at concentrations of 2.50 and 5.00 mg/mL were combined into each Erlenmeyer flask (50 mL). The study was done in triplicate. The Thy/PVA nanoparticles were tested at 1.25 mg/mL (MIC) and 2.5 mg/mL (MBC). Blank nanoparticles in Tween 20 solution were used as a control. All the flasks were incubated at 37 °C and agitated at 1×*g* in an incubator shaker. Every 6 h, during time period of 48 h, 500 μL of culture broth were withdrawn aseptically. The growth of *Y. enterocolitica* was evaluated spectrophotometrically using a microplate reader (Varioskan LUX; Thermo Fisher Scientific) at 600 nm. Sterile medium with Thy/PVA nanoparticles was used as a control. The growth curves were plotted as absorbance (*A*) at 600 nm *versus* incubation time (*t*).

### Antimicrobial efficacy of Thy/PVA nanoparticles

#### Food model

The antimicrobial efficacy of Thy/PVA nanoparticle solution was evaluated on blueberries according to a method described previously (*19*). Blueberries (Berries Paradise, Guadalajara, Mexico) were purchased from a local supermarket in April 2019 and used for this study 11 days before the expiration date printed on the packaging. They were stored at 4 °C prior to use and washed thoroughly with tap water. Then, *Y. enterocolitica* was inoculated by immersing the blueberries in 10 mL of freshly prepared bacterial inoculum for 60 min at 25 °C. The thymol nanoparticle solution was prepared at the concentration of 5 mg/mL (MBC for *Y. enterocolitica*). The blueberries were immersed in 10 mL of thymol nanoparticle solution for 20 min. The blueberries were finally rinsed with sterile distilled water. Tween 20 solution (20 %; *V*/*V*) with blank nanoparticles was set as a control.

#### Bacterial load

Initially, 100 g of blueberries were placed on a sterile Petri dish for a duration of 5 days. The Petri dishes were sealed with parafilm tape to prevent the contamination from other sources and kept at 15 °C. The study was done in triplicate. The blueberries were sampled on daily basis. The morphology of the blueberries was observed. To determine the bacterial load of *Y. enterocolitica*, 250 mL of sterile peptone water (Oxoid, Basingstoke, UK) were added to 25 g of the sample, which was then homogenized using a stomacher (Seward 80, London, UK). Then, 1 mL of crushed sample was serially diluted with sterile peptone water until the colony counts fell within the appropriate range, which is 30–300 colonies per plate. Next, 100 µL of the diluents were spread on MacConkey agar (Oxoid, Basingstoke, UK) plates with a spreader. The plates were placed in an incubator for 48 h and temperature was set at 37 °C. The number of colonies was observed under colony counter (Stuart SC6; Cole-Parmer, St Neots, UK). The experiment was done in triplicate. The results were presented as logarithm of the number of the viable cells (CFU/mL) *versus* incubation time (*t*). Then, Student’s *t*-test was performed using Microsoft Excel (Microsoft Corp., Redmond, WA, USA) to determine the statistical difference between the two test groups.

### Statistical analysis

All the experiments were performed in triplicate, and results were presented as average±standard deviation. Student’s *t*-test was performed to analyse the statistical significance of different test groups in microbiological load study on food models. Statistical experiments and analyses were carried out using the software STATISTICA, v. 7.1 (StatSoft, Tulsa, OK, USA) (*20*).

## RESULTS AND DISCUSSION

Nanoparticle-based drug delivery systems are widely used, especially in preventing postharvest microbial growth on food. This system promises excellent bioavailability, good encapsulation efficiency, controlled chemical release and low toxicity level. The chemical compatibility between the test drug and polymeric encapsulant is a key to a successful nanoparticle delivery system (*21*). PVA was selected in this study because it is an FDA-approved polymer that can be used in contact with food (*22*).

In this study, the size and morphology of Thy/PVA nanoparticles were characterized *via* transmission electron microscope (TEM). The average diameter of thymol nanoparticles was (84.7±11.2) nm (data not shown). Besides, thymol nanoparticles have spherical shapes and smooth surfaces. The result was in line with Zhang *et al.* (*17*), who reported thymol-loaded zein nanoparticles with spherical shape and smooth surface. No sign of agglomeration of nanoparticles was observed based on the electron microscopy analysis. Thymol is a hydrophobic compound, while PVA is a water-soluble polymer that contains a vinyl group (*23*). Pluronic F-127 nonionic detergent was combined in the nanoparticle formulation to avoid the agglomeration of nanoparticles by maintaining their surface energy. The higher the amount of Pluronic F127, the smaller the particle size of nanoparticles (*24*). Thus, the particles formed using a high amount of Pluronic F127 were micelles, because it prevents the coalescence between the nanoparticles, thus improving their stability (*16*). In this study, the amount of Pluronic F127 is sufficient to decrease the size of nanoparticles to a size below 100 nm.

The surface charge of the nanoparticles is important in determining the efficiency and sustainability of the drug delivery process. With DLS, the surface charge of thymol nanoparticles was measured. The result showed that the synthesized nanoparticles had a zeta potential of  −11.73 mV, with a conductivity of 6.655 mS/cm (data not shown). Zeta potential is the electrokinetic potential retained by a molecule at the shear plane of a colloid particle that moves under an electric field (*15*). The stability of the nanoparticles depends on the total potential energy. Therefore, the magnitude of the zeta potential indicates the stability of a nanoparticle system. High repulsion energy of particles with a large negative or positive zeta potential value prevents the particles from agglomeration (*16*). The high zeta potential value of the nanoparticles was justified with the TEM observation.

In total, the encapsulation efficiency of Thy/PVA nanoparticles was 64.99 % (data not shown). The high encapsulation efficiency showed that PVA was suitable for the encapsulation of thymol. The encapsulation efficiency was notably higher than in the previous studies, *e.g.* reported by Li *et al.* (*25*) and McClements (*15*). The type and amount of polymeric matrix could influence the encapsulation efficiency. Besides, the high encapsulation efficiency is also due to the optimal stirring speed during nanoparticle preparation (*24*, *25*). High stirring speed creates high shear stress that causes viscous droplet dispersion. In addition, when the encapsulant concentration increased, the encapsulation efficiency also increased.

**Fig. S1:**
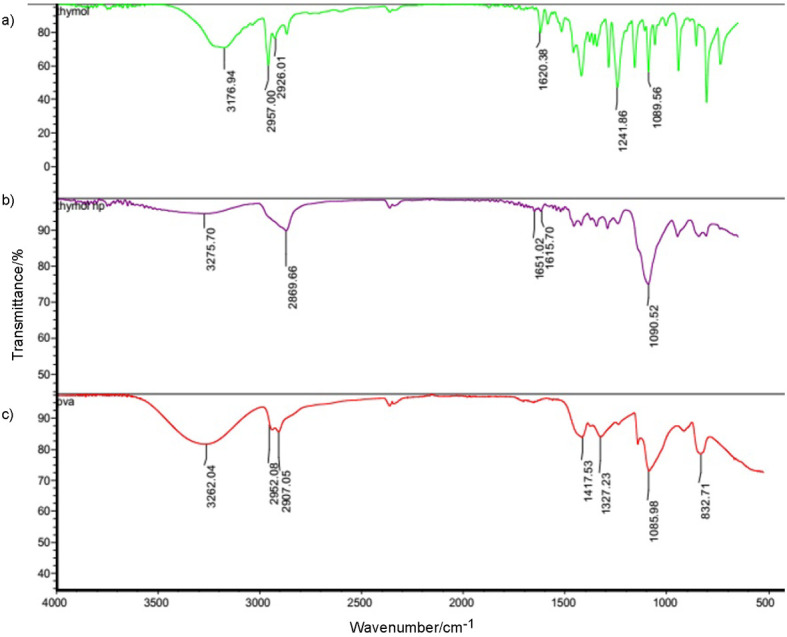
FTIR spectrum for: a) thymol, b) thymol nanoparticles, and c) polyvinyl alcohol

The chemical interactions and functional groups of thymol, thymol nanoparticles and PVA were studied using FTIR spectroscopy (Fig. S1). In the FTIR spectrum of thymol, characteristic absorptions were observed at 3176 cm^-1^ (-OH stretching), 2957 and 2926 cm^-1^ (-CH stretching), and 1620 cm^-1^ (aromatic C=C stretching) (*26*, *27*). On the other hand, PVA showed absorptions at 3262 cm^-1^ (-OH stretching), 2952 and 2907 cm^-1^ (-CH stretching) and 1417 and 1085 cm^-1^ (-C-O- stretching) (*28*). After the nanoencapsulation, thymol nanoparticles showed absorptions at 3275 cm^-1^ due to OH stretching (*29*). The absorption was shifted, which may be due to hydrogen-bound interactions between thymol and PVA. In addition, -CH stretching was observed at 2869 cm^-1^, aromatic C=C stretching at 1651 cm^-1^, and -C-O stretching at 1090 cm^-1^ (Fig. S1). The smaller intensity of absorption was observed in the spectrum of thymol nanoparticles, which may be attributed to a low concentration of thymol in the nanoparticles. The FTIR spectra proved that thymol was successfully encapsulated into the PVA matrix.

**Fig. 1:**
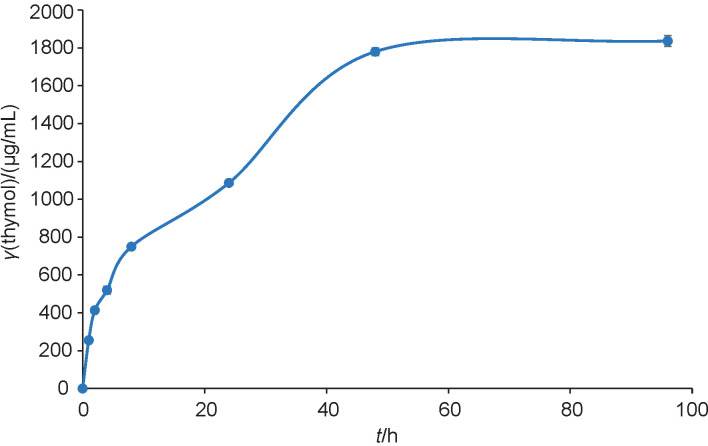
The release of thymol from polyvinyl alcohol nanoparticles

Fig. 1 shows the thymol release pattern from PVA nanoparticles for 96 h. Overall, an initial burst release was observed in the first hour of the experiment. The burst release phenomenon is important for providing sufficient thymol to the food system to inhibit bacterial growth. The rapid burst release of thymol was caused by its rapid diffusion and desorption from the surface of the PVA nanoparticles (*30*). After that, thymol release was slow and gradual, with an average amount of 44.1 μg/mL thymol released per hour. The release was in accordance with the first order of kinetics, where (74.9±5.4) % of thymol was released into the test medium. These results showed the excellent drug carrier properties of the PVA. The sustainable release of thymol was due to the gradual swelling of the nanoparticles when they were exposed to the test medium. A similar trend was reported by Martins *et al.* (*30*). They reported that thymol showed a rapid burst release from the nanofibrous material in the first 2 h, then continued by slow and gradual release until equilibrium. It is worth mentioning that PVA nanoparticles have a high surface to volume ratio and porosity, making them excellent in drug delivery. This characteristic allows the chemical to enhance its drug loading capacity and delivery (*16*). The release of thymol reached a plateau at 48 h. PVA was successfully used to improve the shelf life of the nanoparticles. The drug release pattern proved that PVA was an excellent encapsulant polymer for thymol.

Kirby-Bauer test was conducted to screen the antimicrobial spectrum of Thy/PVA nanoparticles. A total of six test bacteria were tested. Overall, both Thy/PVA nanoparticles showed significant antimicrobial activity on both Gram-positive and Gram-negative bacteria (Table 1). There were no halo zones displayed by solvent control and blank nanoparticles on all test bacteria. This event indicated that the inhibitory activity related to the presence of thymol itself. The largest inhibition zone was represented by MRSA ((17.1±0.1) mm), followed by *Yersinia enterocolitica* ((15.1±0.1) mm).

**Table 1:** The antimicrobial activity of Thy/PVA nanoparticles against foodborne bacteria, represented by inhibition zones, MIC and MBC values

	*d*/mm			*γ*/(mg/mL)	
Test microorganism	Thy/PVA nanoparticle	Thymol-free nanoparticle	Positive control	MIC	MBC
Gram-positive bacteria *S. aureus* MRSA *B. cereus*	9.7±0.4 17.1±0.1 7.2±0.2	- - -	20.1±0.2 17.2±0.3 12.1±0.2	5.00 10.00 2.50	10.00 10.00 5.00
Gram-negative bacteria *Y. enterocolitica* *S. typhii* *E. coli*	15.1±0.3 11.1±0.1 6.4±0.2	- - -	20.2±0.2 21.2±0.4 11.2±0.4	1.25 2.50 2.50	2.50 10.00 5.00

-=no inhibition zone

The quantitative analysis of antimicrobial efficiency for Thy/PVA nanoparticles was done using broth microdilution assay. Generally, Thy/PVA nanoparticles exhibited significant microbicidal activity on foodborne bacteria. A wide range of MICs were observed, ranging from 1.25 to 10.00 mg/mL. The wide range of MICs signified diverse susceptibility of test bacteria to the nanoparticles. Generally, a notable difference was observed between the MIC and MBC of Thy/PVA nanoparticles on all test bacteria, except MRSA. The antibacterial efficiency of thymol nanoparticles on bacteria was in accordance with the concentration. A higher concentration of Thy/PVA nanoparticle was required to allow a killing effect on the test bacteria (MBC), instead of inhibiting the growth (MIC). A notably low MIC and MBC were also observed against *Y. enterocolitica* (Table 1).

**Fig. 2:**
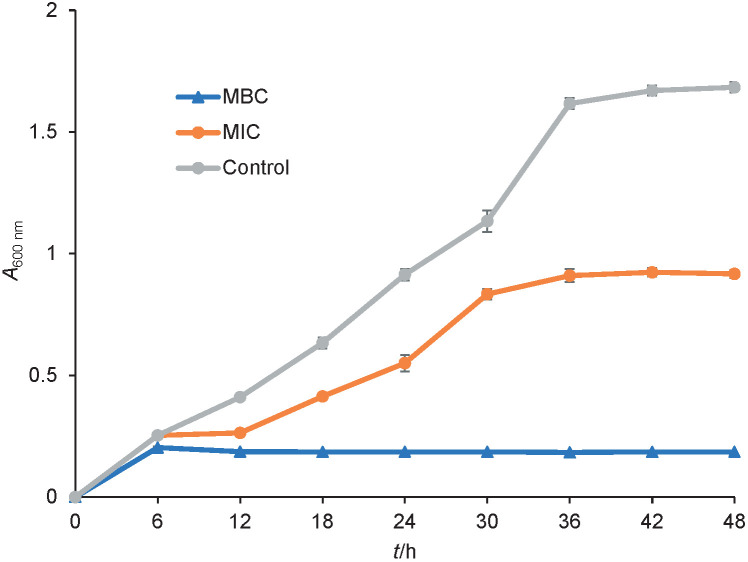
The growth curve of *Yersinia enterocolitica* when exposed to different concentrations of thymol nanoparticles (MIC and MBC), and control (without thymol nanoparticles)

Growth curve study was carried out to study the killing capability of the developed nanoparticles. The investigation was performed on *Y. enterocolitica* using its low MIC and MBC as a reference. Absorbance was measured, which represents the turbidity and mirrors the growth of the bacterium in the broth medium (*18*). In general, the control growth curve showed three distinct growth phases: lag phase, exponential phase, and stationary phase (Fig. 2). Tween 20 solution, which was used to dissolve the Thy/PVA nanoparticle, did not have any inhibitory effect on the growth of *Y. enterocolitica*. In general, the result was in accordance with broth microdilution assay, where the killing efficiency of 99.9 % was not accomplished at MIC. Thy/PVA nanoparticle concentration was not adequate to kill the bacterial cells. The growth curve showed prolonged lag phase and the stationary phase was attained at 30 h. However, the absorbance obtained for MIC was notably lower than for blank nanoparticle control. At MBC, killing 99.9 % of bacterial cells was recorded during the study period. No significant exponential growth of *Y. enterocolitica* was monitored when exposed to thymol nanoparticle at a concentration of MBC, so it was concluded that the nanoparticles were efficient in killing foodborne *Y. enterocolitica*.

Similar observations, *i.e.* a significant antimicrobial activity of thymol against *S. aureus* were previously reported (*31*). Thymol disrupts the outer and inner membrane of bacteria and affects cellular activities and functions of bacterial cells (*12*). Apart from causing serious healthcare-related infections, MRSA has been known as a source of foodborne diseases in the USA over the past three decades (*32*). *Y. enterocolitica* is a causal agent of yersiniosis, clustered as a zoonotic bacterium. It usually triggers a sporadic type of infection (*5*). It is believed that the most common transmission method for *Y. enterocolitica* is through contaminated food *via* the faecal-oral route (*33*). Moreover, imported fruits, including berries, are related to the growth risk of infections by *Y. enterocolitica* (*5*). *S. typhi* is also an important zoonotic bacterium responsible for an extensive worldwide burden of gastroenteritis. This species has been involved in foodborne outbreaks in Australia, with 92 % cases (*34*). The small size of nanoparticles enhances the penetration of thymol into the bacterial cells, thus improving the antimicrobial performance (*35*).

**Fig. S2:**
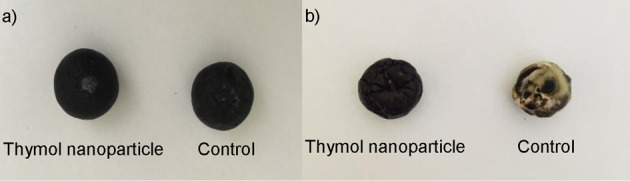
The morphologies of the contaminated blueberries: a) before and b) after the treatment with thymol nanoparticles

The antimicrobial efficacy of Thy/PVA nanoparticles was finally examined in food models as a postharvest treatment. *Y. enterocolitica* causes significant loss of blueberries. Blueberries are fruits with high visual appeal and nutritional value. However, they are very susceptible to microbial infection during the postharvest storage period (*36*). The treatment with thymol nanoparticles maintained the visual quality of blueberries (Fig. S2). Apparent bacterial growth was observed on blueberries treated with nanoparticle control. The decay of blueberries was significantly reduced. Besides, skin colour is an important factor that affects the visual appearance of blueberries (*37*). The treatment with thymol nanoparticles also maintained the skin colour of blueberries.

**Fig. 3:**
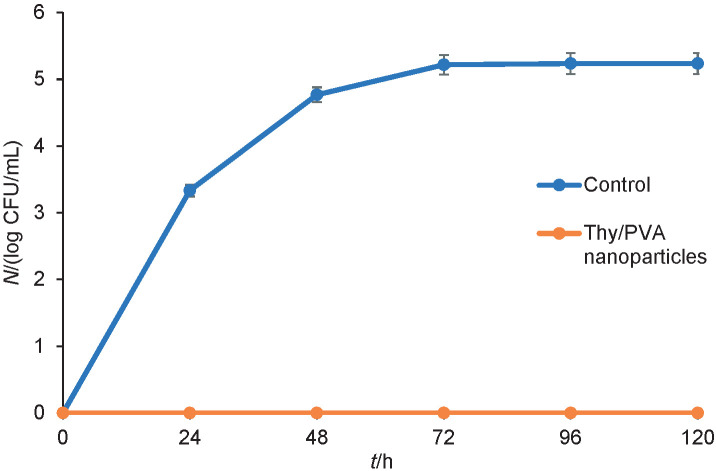
The treatment of blueberries with thymol nanoparticles significantly reduced the bacterial load of *Yersinia enterocolitica* (p≤0.05). The bacterial loads on blueberries treated with the nanoparticles were too few to count (TFTC) throughout the whole experimental period

The treatment of blueberries with thymol nanoparticles also significantly reduced the bacterial load of *Y. enterocolitica* (Fig. 3) which were too few to count (TFTC) throughout the whole experiment. The treatment caused a 100 % reduction of bacterial load on the blueberries up to 5 days. By comparing to nanoparticle control, a significant difference in bacterial load was observed from day 1 (p≤0.05). The fruit coating consisting of Thy/PVA nanoparticles significantly prolonged the storage period of blueberries by reducing the bacterial load that causes significant postharvest quality deterioration. The result was in consensus with Sun *et al.* (*38*). The integration of 0.5 % *trans*-cinnamaldehyde essential oil and chitosan coating on blueberries provided effective protection against *Escherichia coli* and *Penicillium digitatum* at 10 °C for 7 days. Besides, it also protected the fruits from softening. A previous study by Medina *et al.* (*39*) successfully developed chitosan thymol nanoparticle protein films. Chitosan thymol nanoparticles showed good antimicrobial activity for preservation of fresh fruits, and they also act as water vapour barrier when the films are applied on fresh fruits. Sáez-Orviz *et al.* (*40*) also developed thymol nanoparticles for food application using polylactic acid. The gelatine film with these nanoparticles showed high transparency and excellent antimicrobial activity. Both studies are in agreement with the present study.

## CONCLUSIONS

A novel nanoparticle system was successfully developed with thymol, and polyvinyl alcohol (PVA) as encapsulant. The nanoparticles exhibited a sustained release of thymol for 48 h. Thymol nanoparticles showed significant inhibitory activities against both Gram-positive and -negative foodborne bacteria. The nanoparticles also successfully reduced the bacterial load of *Yersinia enterocolitica* on blueberries. Thy/PVA nanoparticles can be potentially used as a postharvest sanitizer for fruits and vegetables, especially blueberries. The application of these sanitizers could improve the microbiological quality of fruits and vegetables, and thus prevent foodborne infections. Further investigations should be conducted to compare the efficacy of Thy/PVA nanoparticles with nonencapsulated thymol.
